# Galectin-10 Expression in Placentas of Women with Gestational Diabetes

**DOI:** 10.3390/cimb45110554

**Published:** 2023-11-02

**Authors:** Christina Buschmann, Laura Unverdorben, Julia Knabl, Stefan Hutter, Sarah Meister, Susanne Beyer, Maximiliane Burgmann, Lucia Keilmann, Alaleh Zati zehni, Elisa Schmoeckel, Mirjana Kessler, Udo Jeschke, Sven Mahner, Thomas Kolben, Franziska Ganster, Alexander Burges

**Affiliations:** 1 Department of Obstetrics and Gynecology, University Hospital, LMU Munich, 81377 Munich, Germany; 2 Department of Pathology, University Hospital, LMU Munich, 81377 Munich, Germany; 3 Department of Obstetrics and Gynecology, University Hospital Augsburg, 86156 Augsburg, Germany

**Keywords:** galectin-10, gestational diabetes mellitus, pregnancy

## Abstract

Galectins are known to play an important role in immunoregulatory processes and autoimmune diseases. Galectin-10 is a cytoplasmic protein of human eosinophils and is involved in various eosinophilic diseases. Since increased galectin expression is already detected in the placentas of mothers with gestational diabetes mellitus (GDM), this study focuses on the specific role of galectin-10 and hints at consequences for the diagnosis and therapeutic options of GDM. It is hypothesized that the difference in galectin-10 expression will raise the pathophysiological understanding of gestational diabetes. The study population consists of 80 women: 40 healthy mothers and 40 women suffering from gestational diabetes mellitus. The expression of galectin-10 was analyzed in the syncytiotrophoblast (SCT) and the decidua of the placenta via immunohistochemistry and immunofluorescence double staining. The immunoreactivity score (IRS) was used for evaluation. The results in this study were significant for an overexpression of galectin-10 in GDM placentas compared with the control group. The syncytiotrophoblast showed overexpression in the nucleus and the cytoplasm, whereas expression of galectin-10 in the decidua was significant in the cytoplasm only. This study identified the expression changes in galectin-10 in placental tissue between healthy and GDM mothers and intensified the understanding of gestational diabetes. Assuming that gestational diabetes mellitus is involved in inflammatory processes, galectin-10 might play a role in the development and maintenance of GDM. Further investigation is required to strengthen these findings.

## 1. Introduction

Gestational diabetes mellitus (GDM) is a growing health concern worldwide. GDM is defined as a glucose tolerance disorder that arises during pregnancy [1]. 

For glucose control, insulin secretion is necessary to be increased during pregnancy. Women who are suffering from GDM are not able to increase pancreatic insulin production for some reason. Consequently, higher levels of blood sugar can be measured. 

It can be assumed that the prevalence of diabetes mellitus and GDM will increase within the next years. 

Predisposed conditions are genetic, social, and environmental factors such as obesity and overweight, maternal age above 24 years, and family history of diabetes type 2 or GDM [2].

Suffering from GDM can affect the fetus and the mother.

Sequelae in the fetus are macrosomia and medical complications after delivery including diabetic embryopathy, infant respiratory distress syndrome, cardiomyopathy, hypoglycemia, hypocalcemia, hypomagnesemia, polycythemia, hyperviscosity, and life-long increased risk of glucose intolerance and obesity.

On the maternal side, a higher long-term risk exists for developing preeclampsia, metabolic syndrome, hypertension, urinary tract infections, type 2 diabetes, and delivery via cesarean section due to fetal macrosomia. Knowledge about maternal and fetal consequences implies risk-adjusted antepartum surveillance, delivery planning, intrapartum glucose management, and postpartum care [1,3].

Screening and diagnosing diabetes mellitus are not uniform worldwide. This has led to under diagnosis and undermanagement of the disease.

To detect GDM, an oral glucose tolerance test should be performed between 24 and 28 weeks of pregnancy with a glucose-containing drink. Blood glucose measurements either at baseline, one hour, or two hours above the set limits indicate the diagnosis of GDM [1].

Galectins are proteins characterized by an individual amino acid sequence called carbohydrate recognition domain (CRD) in the carbohydrate-binding domain and their affinity to ß-galactoside sugars. The growing protein family of galectins is classified by their molecular structure and their different number of CRDs [4,5,6].

By now, 16 members of the family have been identified so far. Extensive research into the significance and function of galectins has been performed over the last few years. Each galectin fulfills different purposes depending on the tissue and the location.

Galectins play key roles as modulators of immune surveillance, apoptosis, cell adhesion, chemotaxis, and synthesis of inflammatory mediators such as cytokines, nitric oxide, and prostaglandins. Furthermore, they might trigger opposite effects toward the same cell type depending on the carbohydrate characterization [6]. The function of the galectin members within different processes varies a lot. They are involved in intracellular signaling and communication and affect cell proliferation and survival. 

As galectins influence immunoregulatory processes, autoimmune diseases, and cancer, they are potentially interesting targets in anti-inflammatory and anticancer therapies. For this, more understanding of the mechanisms of cell modulation by galectins is required before galectin-based therapeutic strategies can be realized [7].

Increased expression of members of the galectin family has already been identified in the cytotrophoblast and syncytiotrophoblast of the placenta. Angiogenesis and immunomodulation influence implantation, placentation, and fetal–maternal tolerance. Overexpression of galectin-1 and galectin-3 is described in preeclamptic placentas of the third trimester and in placentas of women suffering from hemolysis, elevated liver enzymes, and low platelets (HELLP). In the uterus, galectin-1 is mainly located in the endometrial stroma and galectin-3 is found in the endometrial glandular epithelium. Furthermore, the expression of galectin-1 and galectin-3 depends on the various phases of the menstruation cycle [8]. Furthermore, an upregulation of galectin-2 could be demonstrated in the decidua of placentas with GDM. Galectin-2 is known to modulate pro- and anti-inflammatory actions. Previous studies pointed out a decreased level of galectin-2 in the placental tissue of miscarriages, preeclampsia, and male cases of intrauterine growth restriction (IUGR) [9].

Galectin-10 is a cytoplasmic protein of human eosinophils and belongs to the lectin family. Galectin-10 forms Charcot–Leyden crystals, which are observed in various eosinophilic diseases and can act as a biomarker for disease activity, diagnosis, and treatment effectiveness in eosinophilic diseases like asthma, eosinophilic esophagitis, rhinitis, and atopic dermatitis. Galectin-10 is exclusively present in eosinophils in the human body [10,11]. Interestingly, extracellular release is mediated by extracellular trap cell death, an active cell death program. Determining eosinophilic inflammation via blood eosinophilic count is not precise, so the identification of blood biomarkers is an approach to improve the accuracy of diagnosis. Galectin-10 might be one of the potential proinflammatory biomarkers [10]. Structurally, it forms a homo-dimer in the face-to-face orientation and monosaccharides bind to the carbohydrate recognition site [11]. Charcot–Leyden crystals have also been detected in basophiles and macrophages. In the human body, this protein can spontaneously form Charcot–Leyden crystals in lymphocytes, but its role is not fully understood so far. The location of galectin-10 in the nucleus suggests the involvement in gene expression functions [12].

The role of galectin-10 in pregnancy is not described in the literature so far.

For this prospective study, we investigated the expression of galectin-10. Assuming that GDM is an inflammatory disease, the objective of this preliminary study focuses on the expression changes in galectin-10 in placental tissue between healthy and GDM mothers. 

## 2. Materials and Methods

### 2.1. Study Design

Approval for this study was obtained by the Ethics Committee of the faculty of the LMU of Munich on 26 January 2010 with Approval No. 337-06. A total of 80 pregnant women were included in this study after obtaining written consent. The women were divided into 40 healthy women (control group) and 40 women suffering from GDM (case group) between 2013 and 2015. Fetal gender was balanced in both groups. Inclusion criteria were a maternal age above 18 years, singleton pregnancies, diagnosed GDM via oral glucose tolerant test (oGTT) for the case group, or exclusion of GDM via oGTT for the control group. Exclusion criteria were maternal age under 18, multiple pregnancies, pregnancy-related diseases, and no capacity for consent. 

Diagnosis criteria for GDM were based on the criteria of the German Society of Diabetes Mellitus. All participants underwent oGTT between 24 and 28 weeks of pregnancy. One pathological measurement identified GDM: fasting glucose serum level >92 mg/dL, after one hour >180 mg/dL, and after two hours >153 mg/dL.

Moreover, clinical data like maternal body mass index (BMI), birth weight, insulin therapy, and birth mode were collected in both groups.

For each case, tissue samples measuring 2 × 2 × 2 cm^3^ of a central placenta cotyledon were obtained directly after delivery containing maternal decidua, fetal SCT, and amniotic epithelia. For further analyses, samples were fixated in a 4% buffered formalin solution and embedded in paraffin. 

### 2.2. Immunohistochemical Staining

The immunohistochemical staining procedure was based on a detailed protocol published by Hutter et al. [13]. After removing the paraffin from the placenta tissue samples in a Roticlear bath (Carl Roth, Karlsruhe, Germany), endogenous peroxidase activity was blocked with a 3% H2O2 solution. In the next step, high-pressure sodium citrate (pH 6.0) treatment was used for demasking the protein epitopes using a blocking solution (ZytoChem Plus HRP Polymer System, Zytomed Systems GmbH, Berlin, Germany). After incubation with primary anti-galectin-10-antibody (polyclonal rabbit IgG, concentrate 0.05 mg/mL, NBP1-89690, Novus Biologicals, Minneapolis, MI, USA), the samples were dissolved in PBS at 1:200 dilution for 16 h, at 4 °C, and then treated with Post Block (Reagent 2, ZytoChem Plus HRP Polymer System mouse/rabbit, Zytomed) for 30 min. Thereafter, the slides were treated with chromogen 3,3′-diaminobenzidine (DAB; Dako; Glostrup, Denmark) to visualize the detection of galectin-10. Positive and negative immunohistochemistry controls were induced with human colon tissue for anti-galectin-10-antibody. Positive controls ensure the viability of the antibody. For the negative controls, anti-rabbit-Ig-containing serum was applied instead of the primary antibody. Mayer’s hemalum was used for counterstaining. 

All samples were evaluated under a Leitz Diaplan microscope using 10-fold and 40-fold objectives.

The semi-quantitative immunoreactivity score (IRS) evaluated the cell staining intensity (0: none; 1: weak; 2: moderate; and 3: strong) and the percentage of positively stained cells (0: no staining; 1: <10% of the cells; 2: 11–50%; 3: 51–80%; and 4: >80). The values were counted in an IRS between 0 and 12 for each slide in four fields per slide in a 40-fold lens. The calculation was as follows: mean staining intensity × percentage = IRS (minimum = 0, maximum = 12) [14]. All samples were evaluated by two different observers counting a minimum of 100 cells. Blinding was applied during the IRS assessment. 

As Image J was used to objectify the IRS in GDM placentas, the values generated were analyzed (ImageJ 1.54d, Wayne Rasband and contributors, National Institutes of Health, Bethesda, MD, USA). Here, significance was shown in the IRS of nuclear SCT (*p* = 0.014) as well as nuclear decidua (*p* = 0.015) and cytoplasmic decidua (*p* = 0.010), which correlates with the values of our IRS results. The overview is provided in Table 1.

### 2.3. Double Immunofluorescence

Cytokeratin 7 (CK7) is a marker for extravillous trophoblast cells and was used in this study for a phenotypical differentiation between the maternal and the fetal cells.

After removing the paraffin and demasking protein epitopes, a blocking solution (Ultra V-Block, Thermo Scientific, Lab Vision, Fremont, CA, USA) was applied for 15 min to avoid certain antigen–antibody interactions.

The samples were incubated with a primary antibody mixture following treatment with a secondary fluorescent antibody mixture for 30 min. For nuclear counterstaining, the slides were treated with mounting buffer (Vector Laboratories, Burlingame, CA, USA), which contains DAPI. The sample was analyzed with the fluorescent Axioskop photomicrocope (Zeiss, Oberkochen, Germany) using a digital Axiocam camera system (Zeiss, Oberkochen, Germany) under a 63-fold objective. An overview of antibodies and working dilution is provided in Table 2.

### 2.4. Statistical Analysis

Statistical analysis was performed by using IBM SPSS Statistics (Version 26 for MAC, Armonk, NY; USA). The data were analyzed using non-parametric t-tests and the Mann–Whitney U test, as well as the Kruskal–Wallis test, which was used for the analysis of continuous variables. Multivariate analysis was performed by using a linear regression model to analyze the influence of gender and maternal BMI on the galectin-10 expression and to identify these factors for to analyze confounding. A *p*-value of <0.05 was considered to be significant.

## 3. Results

### 3.1. Study Population

In this study, 80 pregnant women were included and divided into a case group of 40 women (diagnosed with GDM) and a control group of 40 women (healthy). Newborns were delivered maturely after the 37th week of pregnancy. Fetal gender was equal with 20 males and 20 females in each group. Considering maternal BMI as a continuous variable, BMI was significantly higher in the case group: 28.13 kg/m^2^ compared with 23.35 kg/m^2^ (*p* = 0.002).

Insulin therapy was required in 30/40 (75.0%) of the GDM patients. Furthermore, average birth weight showed significance and was higher in the case group (3611 g) compared with the control group with 3317 g (*p* = 0.013). Table 3 represents these data.

We analyzed the expression of galectin-10 in the SCT (fetal part) and the decidua (maternal part) of the placenta samples. IRS was used for evaluation. An overview of the IRS is provided in Table 4.

### 3.2. Galectin-10 Is Increased in Fetal SCT and Decidua of GDM Placentas

**Figure 1:**
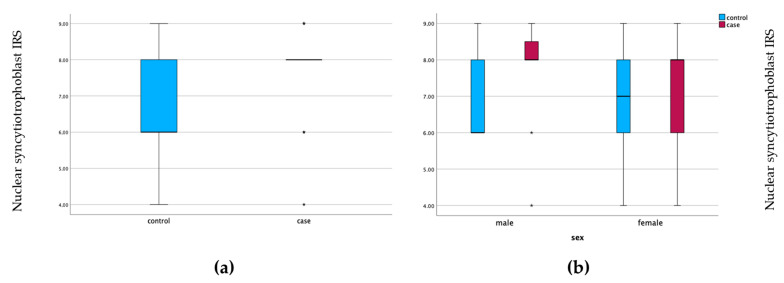
Galectin-10 expression in nuclear syncytiotrophoblast of GDM placentas (case) and healthy controls (**a**). Gender-specific galectin-10 expression. Boxplots show the IRS for galectin-10 expression by fetal gender in the nuclear syncytiotrophoblast (**b**).

Our results indicate that the IRS of galectin-10 is increased in the nuclear SCT of GDM placentas compared with the control group and is statistically significant (*p* = 0.015). The cytoplasmic expression in the SCT was not significantly altered. (*p* = 0.763). This is underlined in Figure 1.

**Figure 2:**
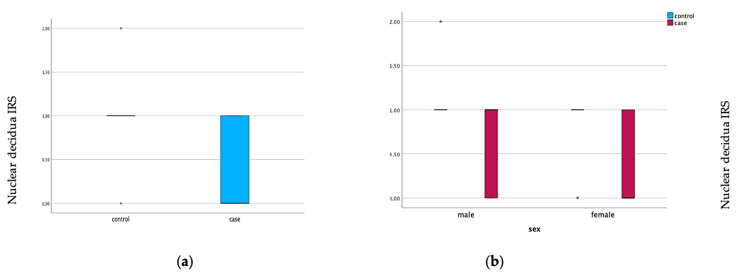
Galectin-10 expression in nuclear decidua of GDM placentas (case) and healthy controls (**a**). Gender-specific galectin-10 expression. Boxplots show the IRS for galectin-10 expression in nuclear decidua (**b**).

**Figure 3:**
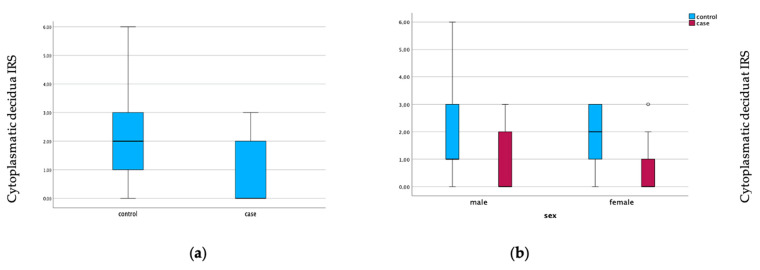
Galectin-10 expression in the cytoplasmic decidua of GDM placentas (case) and healthy controls (**a**). Gender-specific galectin-10 expression. Boxplots show the IRS for galectin-10 in cytoplasmic decidua (**b**).

**Figure 4:**
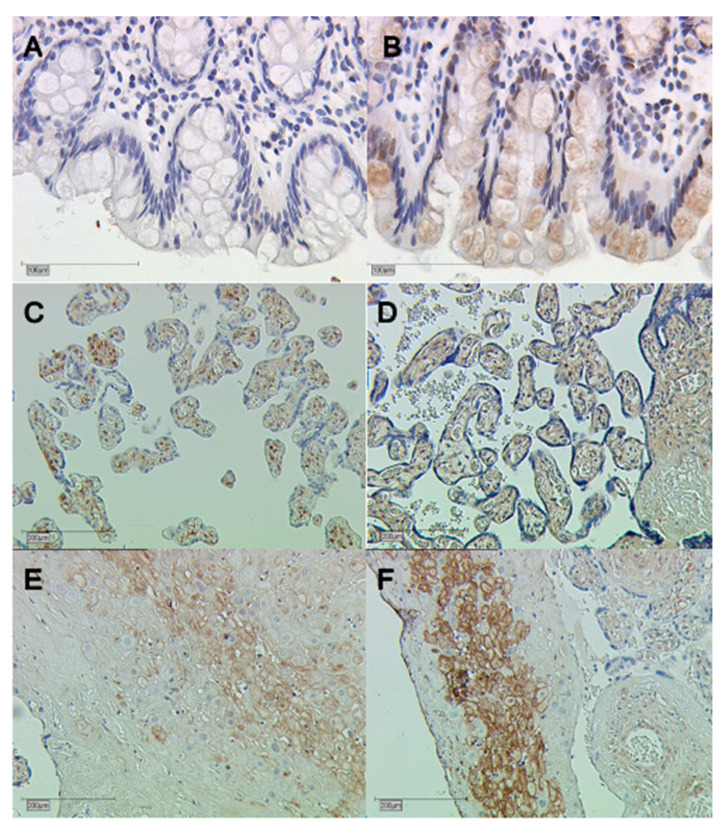
Immunohistochemical staining of galectin-10. (**A**) negative control of human colon tissue; (**B**) positive control of human colon tissue; (**C**) galectin-10 staining in the syncytiotrophoblast of control placentas; (**D**) galectin-10 staining in the syncytiotrophoblast of GDM placentas; (**E**) galectin-10 staining in the decidua of control placentas; and (**F**) galectin-10 staining in the decidua of GDM placentas.

Furthermore, the expression of galectin-10 was elevated in the decidua of GDM placentas compared with healthy placentas. Here, the cytoplasmic expression (*p* = 0.004) and the nuclear expression were significantly elevated (*p* = 0.001). The expression of galectin-10 is illustrated in Figure 2 and Figure 3. Representative pictures of the immunohistochemical staining are shown in Figure 4. The scale bar equals 100 µm (Figure 4A,B) and 200 µm (Figure 4C–F).

### 3.3. Galectin-10 Overexpression Visualized Using Immunofluorescence Double Staining

**Figure 5:**
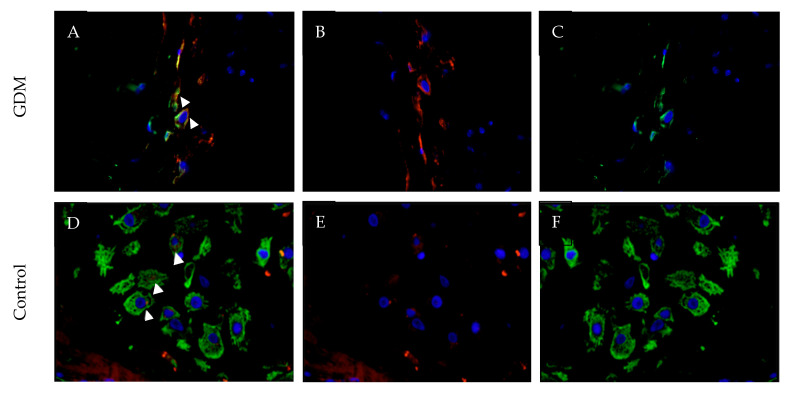
Double immunofluorescence phenotyping of decidual cells of the placenta in 40× magnification. Nuclei are stained with DAPI (blue). Galectin-10 is stained red. CK7 is stained green, marking the extravillous trophoblast (EVT). (**A**) Galectin-10 expression in GDM placenta. Nuclei are stained with DAPI (blue) and arrows indicate the merging expression of CK7 and galectin-10. (**B**) Galectin-10 expression in GDM placenta. Nuclei are stained with DAPI (blue), galectin-10 is stained red; and (**C**) galectin-10 expression in GDM placenta. Nuclei are stained with DAPI (blue) and CK7 is stained green. (**D**) Galectin-10 expression in the control group. Nuclei are stained with DAPI (blue) and arrows indicate the merging expression of CK7 and galectin-10. (**E**) Galectin-10 expression in the control group. Nuclei are stained with DAPI (blue) and galectin-10 is stained red. (**F**) Galectin-10 expression in the control group. Nuclei are stained with DAPI (blue) and CK7 is stained green.

To visualize the expression of galectin-10, the immunofluorescence double staining technique was used. Cytokeratin 7 (CK7) is a marker for extravillous trophoblast cells (EVTs) and was used for the differentiation between maternal and fetal cells [15]. The microscopic evaluation identified co-expression of CK7 and galectin-10 in the same cell type, thus confirming that overexpression of galectin-10 could be represented in the extravillous trophoblast (Figure 5).

### 3.4. Multiple Regression Analysis

Our results showed no significant difference in galectin-10 expression in the SCT (*p* = 0.073) and the decidua (*p* = 0.298) within the gender group. Maternal BMI was additionally analyzed with the linear regression model. No significant difference could be pointed out for the SCT (*p* = 0.0). 

Therefore, in this study, fetal gender and maternal BMI did not seem to be a confounder of the differences in placental galectin-10 expression. 

## 4. Discussion

The global prevalence of diabetes is high and the frequency of GDM is increasing. Wild et al. estimate the total number of people with diabetes to be 366 million worldwide by 2030. The prevalence of diabetes is higher in men than in women [16]. The World Health Organization established criteria to diagnose GDM as follows: fasting plasma glucose = 5.1–6.9 mmol/L (92–125 mg/dL), 1 h post 75 g oral glucose load > = 10.0 mmol/L (180 mg/dL), and 2 h post 75 g oral glucose load 8.5–11.0 mmol/L (153–199 mg/dL) [17]. Treating GDM is important to reduce the risks of preeclampsia, shoulder dystocia, macrosomia, and glucose dysregulation in newborns [18].

Galectins are known to play a key role in inflammatory and autoimmune processes including diabetes mellitus and GDM.

This study focuses on the potential role of galectin-10 in the placentas of women suffering from GDM. At present, the expression of galectin-10 in placental tissue has not been described so far. The expression of galectin-10 was analyzed in the SCT, representing the fetal part, and the decidua, representing the maternal part, of the placenta. Furthermore, we differentiated the expression of galectin-10 in the nucleus and the cytoplasm. The results detected a significant overexpression in the nuclear and cytoplasmic SCT and in the cytoplasmic decidua of GDM placentas compared with the healthy control group. 

As fetal gender and maternal weight are assumed to be risk factors for developing GDM, we performed a linear regression analysis and could rule out those parameters as potential confounders for the expression of placental galectin-10 in this study. 

Previous studies already displayed a difference in the expression of galectins between GDM placentas and healthy placentas. Galectin-4 is considered to be a proinflammatory factor in chronic bowel diseases. As galectin-4 stimulates interleukin-6, another proinflammatory cytokine, this interaction could indicate an immunological impact within the inflammation process. Overexpression of galectin-4 could be identified in GDM placentas [19]. Blois et al. described increased levels of galectin-1 locally in the placenta and peripherally in the circulation of women with GDM. Peripheral overexpression of galectin-1 was observed in the second and third trimester of pregnancy. 

Dysregulation of galectin-1 can also be connected with pregnancy-related diseases, even in the early stages of pregnancy [20]. Galectin-1 was found to be located in the endometrial stroma. Furthermore, an overexpression of galectin-1 was detected in cases of chorioamnionitis and preeclampsia, underlining its role in inflammation [21]. 

Galectin-3 is the only known chimera-type galectin. A weak expression of galectin-3 was found in healthy first-trimester placentas, whereas an elevated level of galectin-3 is correlated with reduced trophoblast invasion and development of pregnancy-related diseases like preeclampsia and HELLP syndrome [22]. 

A protective effect of galectin-2 was also described by Paclik et al. Here, a mouse model was used to describe the therapeutic effect of galectin-2 in acute and chronic colitis cases. As galectin-2 might play a role in angiogenesis, lack of galectin-2 in the placenta may contribute to miscarriages. This underlines the protective effect of galectin-2 in various tissues [22,23]. 

Another member of the galectin family, galectin-13, is structurally similar to galectin-10, which was investigated in this study. Galectin-13 was identified to be bound to annexin II and beta/gamma actin in placental tissue and fetal hepatic cells. Due to its specific binding to annexin II, galectin-13 is assumed to be secreted to the outer surface in actin and annexin II-containing microvesicles via exocytosis [24]. Galectin-13 is also known as placenta protein 13 (PP-13). It is supposed to exert immunobiological and hemostatic functions at the fetal–maternal interface and in the development of the placenta. Galectin-13 is decreased in the SCT, in the nuclei of SCT, and in trophoblast cells, as well as in the extravillous trophoblast cells of placentas with GDM compared with healthy controls, which is in coherence with the assumption that it plays an anti-inflammatory role in this process. Galectin-13 might act as a chemokine, inducing the formation of zones of necrosis by activating interleukin 1alpha and interleukin 6 of immune cells in the decidua of the placenta. Additionally, levels of galectin-13 in serum blood were decreased in women suffering from GDM. In summary, the lack of galectin-13 can lead to inflammation and an adverse outcome in pregnancy because of inflammatory effects [25]. In accordance with the fact that galectin-10 is associated with eosinophilic and thus inflammatory diseases and the knowledge about the involvement of other members of the galectin family in pregnancy-related inflammatory processes, galectin-10 was identified as a candidate of interest for this study. Compared with the analysis of galectin-13, the expression of galectin-10 was increased in the placentas from GDM, which suggests a more inflammatory role. 

In addition to galectins, cytokines also act as modulators of the immune system. Pro-inflammatory cytokines like interleukins (IL) have a regulatory function in placental tissue. Keckstein et al. investigated the expression of interleukin-7, -8, and -15 in GDM placentas. Here, the expression of IL-15 was significantly upregulated in the extravillous trophoblast of women with GDM, and the expression of IL-8 was downregulated in the EVTs of GDM placentas. Interleukin-7 was higher expressed in GDM placentas. An upregulation of interleukins in GDM placentas indicated an involvement of cytokines in the maintenance of GDM as a state of inflammation in the placenta. Interestingly, gender-specific differences in the expression patterns could be ruled out in this study [26]. While the function of the different types of cytokines and galectins varies, further investigation is important to rule out the interaction of cytokines and galectin-10 in the placenta within the inflammatory and immune-modulating process of GDM. As a specificity and sensitivity comparison was not performed, it might be conducted in the future. 

To summarize, members of the galectin family play an important role in the process of pregnancies and are differently regulated at the human fetal–maternal interface and in pregnancy-related diseases like GDM, miscarriages, preeclampsia, and IUGR [13,27,28]. 

With galectin-10, this study identified another member of the complex family of galectins, whose expression differs in placental tissue between healthy and GDM mothers. The upregulation of galectin-10 further underlines the involvement of galectins in inflammatory processes and pregnancy-related diseases. As previous studies already illustrated, it might be interesting to investigate the expression patterns of galectin-10 in pregnancy-related diseases besides GDM, such as preeclampsia, HELLP syndrome, and miscarriages, to determine the function of galectin-10 in the development and maintenance of inflammation. Furthermore, a correlation with chemokines and cytokines might be interesting to point out the inflammation level. A limitation of this study is a lack of data concerning the serum levels of galectin-10, which could point out a systematic relevance of changes in galectin-10 expression. 

Moreover, women suffering from diseases like asthma, rhinitis, allergies, and rheumatoid arthritis were not excluded from the study population. These eosinophilic diseases might influence the expression of galectin-10 levels in both groups. 

Additionally, potential biomarkers for predicting the onset of preeclampsia and gestational diabetes before delivery might intensify the results of this study. Chronic villous sampling is not possible as it is known to increase a higher risk of miscarriage.

The study population consists of 80 pregnant women balancing 40 women in a healthy group and 40 women in a group diagnosed with GDM. A limitation concerning the study population is that no sample size calculation was performed. As investigating the expression of galectin-10 in placental tissue was not mentioned in the previous literature, this analysis provides a basis for further investigation.

The extension of the study population and implementation as a multicenter study would be necessary to prove our results.

Obviously, GDM is a rising health concern worldwide with a growing incidence. Structural improvement in diagnostic testing and global access to treatment is necessary to prevent adverse outcomes for mother and child. The knowledge of the regulation mechanisms of galectin-10 in the placental tissue of GDM mothers complements previously known correlations of the galectin expression in GDM and enables additional diagnostic approaches. 

However, this study provides evidence that dysregulation of galectin-10 as an important modulator of inflammatory processes is newly identified in placentas with GDM. 

Further investigation is necessary to understand the complex mechanisms of the galectin group in pregnancy-related diseases in order to implement the use of these proteins in diagnosis and therapy.

## Figures and Tables

**Table 1:** IRS quantification with Image J (imagj.net/ij/download.html, accessed on 1 October 2023).

		GDM			Control		*p*-Value
	Mean	Median	Modus	Mean	Median	Modus	
SCT nuclear	6.1238	7	7	5.2657	6	6	0.014
SCT cytoplasmic	2.1349	2	2	2.2234	2	2	0.752
Decidual nuclear	1.1208	1	1	0.8963	1	1	0.015
Decidual cytoplasmic	1.1657	1	0	1.1241	0	1	0.010

**Table 2:** Antibody features used for immunofluorescence.

Antibody	Dilution	Incubation	Manufacturer
Galectin-10—polyclonal Rabbit IgG	1:200	16 h at 4 °C	Novus Biologicals, Centennial, CO, USA—NBP1-89690
CK7—Clone OVTL Mouse IgG	1:30	16 h at 4 °C	Novocastra, Leica Biosystems, IL, USA—NCL-L-CK7-OVTL
CD31—Clone JC/70A Mouse IgG	1:50	16 h at 4 °C	Abcam, Cambridge, UK—ab9498
Cy-2-labelled goat-anti-rabbit	1:100	30 min at RT	Dianova, Hamburg, Germany—115-226-062
Cy-3-labelled goat-anti-mouse	1:500	30 min at RT	Dianova, Hamburg, Germany—111-165-144

**Table 3:** Demographic and clinical data of the study population.

	GDM		Control		*p*-Value
Gender	n	%	n	%	
Male	20	50.0	20	50.0	
Female	20	50.0	20	50.0	
Maternal BMI prior to pregnancy (kg/m^2^)					
Underweight (<18.5)	0	0	4	10.0	0.116
Normal (18.5–24.9)	16	40.0	25	62.5	0.044
Overweight (25.0–29.9)	10	25.0	3	7.5	0.034
Obese (≥30.0)	12	30.0	5	12.5	0.056
Insulin therapy					
	30	75.0	0	0	
Delivery mode					
Vaginal	27	67.5	32	80.0	0.310
C-section	13	32.5	8	20.0	0.310
Fetal birth weight (g)					
Low birth weight (<3000)	1	2.5	2	5.0	0.556
Normal birth weight (3000–4000)	30	75.0	33	82.5	0.412
High birth weight (>4000)	9	22.5	5	12.5	0.239
Duration of gestation and delivery (weeks)					
Male	39.67 ± 1.30			39.80 ± 1.30	
Female	39.83 ± 1.40			39.75 ± 1.40	

**Table 4:** IRS.

		GDM			Control		*p*-Value
	Mean	Median	SD	Mean	Median	SD	
SCT nuclear	7.609	8	1.222	6.875	6	1.343	0.015
SCT cytoplasmic	2.049	2	0.384	2.073	2	0.412	0.763
Decidual nuclear	0.469	0	0.507	0.974	1	0.362	0.001
Decidual cytoplasmic	0.906	0	1.117	1.974	2	1.267	0.004

## Data Availability

Due to privacy restrictions data is unavailable.

## Abbreviations

BMI	Body mass index
CK7	Cytokeratin 7
cm	Centimeter
dL	Deciliter
EVT	Extravillous trophoblast cell
GDM	Gestational diabetes mellitus
h	Hours
IRS	Immunoreactivity score
IUGR	Intrauterine growth restriction
kg	Kilogram
m	Meter
mmol	Millimole
oGTT	Oral glucose tolerant test
PP-13	Placenta protein 13
SCT	Syncytiotrophoblast

## References

[1] Reece E.A. (2009). Gestational diabetes: The need for a common ground. Lancet.

[2] Lende M. (2020). Gestational Diabetes: Overview with Emphasis on Medical Management. Int. J. Environ. Res. Public. Health.

[3] Schneider S. (2011). Neonatal complications and risk factors among women with gestational diabetes mellitus. Acta Obstet. Gynecol. Scand..

[4] Barondes S.H. (1994). Galectins. Structure and function of a large family of animal lectins. J. Biol. Chem..

[5] Kasai K. (1996). Galecints: A family of animal lectins that decipher glycocodes. J. Biochem..

[6] Rabinovic G.A. (2002). Role of galectins in inflammatory and immunomodulatory processes. Biochim. Biophys. Acta.

[7] Rabinovic G.A. (2007). Functions of cell surface galectin-glycoprotein lattices. Curr. Opin. Struct. Biol..

[8] Jeschke U. (2013). Expression and function of galectins in the endometrium and at the human feto-maternal interface. Placenta.

[9] Hepp P. (2020). Placental Galectin-2 Expression in Gestational Diabetes: A Systematic, Histological Analysis. Int. J. Mol. Sci..

[10] Tomizawa H. (2022). Galectin-10 as a Potential Biomarker for Eosinophilic Diseases. Biomolecules.

[11] Itoh A. (2020). Structures of human galectin-10/monosaccharide complexes demonstrate potential of monosaccharides as effectors in forming Charcot-Leyden crystals. Biochem. Biophys. Res. Commun..

[12] Su J. (2018). A Brief History of Charcot-Leyden Crystal Protein/Galectin-10 Research. Molecules.

[13] Hutter S. (2015). Fetal gender specific expression of tandem-repeat galectins in placental tissue from normally progressed human pregnancies and intrauterine growth restriction (IUGR). Placenta.

[14] Stampfl S. (2009). Immunhistochemical Characterization of specific inflammatory tissue reactions following embolization with four different spherical agents in the minipig kidney model. J. Vasc. Interv. Radiol..

[15] Maldonado-Estrada J. (2004). Evaluation of Cytokeratin 7 as an accurate intracellular marker with which to asess the purity of human placental villous trophoblast cells by flow cytometry. J. Immunol. Methods.

[16] Wild S. (2004). Global prevalence of diabetes: Estimates for the year 2000 and projections for 2030. Diabetes Care.

[17] (2014). Report of World Health Organization Consultation, Diagnostic criteria and classification of hyperglycaemia first detected in pregnancy: A World Health Organization Guideline. Diabetes Res. Clin. Pract..

[18] Hartling L. (2013). Benefits and harms of treating gestational diabetes mellitus: A systematic review and meta-analysis for the U.S. Preventive Services Task Force and the National Institutes of Health Office of Medical Applications of Research. Ann. Intern. Med..

[19] Schrader S. (2022). Overexpression of galectin-4 in placentas of women with gestational diabetes. J. Reprod. Immunol..

[20] Blois S.M. (2014). Getting too sweet: Galectin-1 dysregulation in gestational diabetes mellitus. Mol. Hum. Reprod..

[21] Jeschke U. (2006). Binding of galectin-1 (gal-1) to the Thomsen-Friedenreich (TF) antigen on trophoblast cells and inhibition of proliferation of trophoblast tumor cells in vitro by gal-1 or an anti-TF antibody. Histochem. Cell Biol..

[22] Unverdorben L. (2016). Prototype and Chimera-Type Galectins in Placentas with Spontaneous and Recurrent Miscarriages. Int. J. Mol. Sci..

[23] Paclik D. (2008). Galectin-2 induces apoptosis of lamina propria T lymphocytes and ameliorates acute and chronic experimental colitis in mice. J. Mol. Med..

[24] Than N.G. (2004). Functional analyses of placental protein 13/galectin-13. Eur. J. Biochem..

[25] Unverdorben L. (2015). Galectin-13/PP-13 expression in term placentas of gestational diabetes mellitus pregnancies. Placenta.

[26] Keckstein S. (2020). Sex Specific Expression of Interleukin 7, 8 and 15 in Placentas of Women with Gestational Diabetes. Int. J. Mol. Sci..

[27] Unverdorben L. (2016). Comparative analyses on expression of galectins1-4, 7-10 and 12 in first trimester placenta, decidua and isolated trophoblast cells in vitro. Histol. Histopathol..

[28] Than N.G. (2009). A primate subfamily of galectins expressed at the maternal-fetal interface that promote immune cell death. Proc. Natl. Acad. Sci. USA.

